# Pancreatitis severity in mice with impaired CFTR function but pancreatic sufficiency is mediated via ductal and inflammatory cells‐Not acinar cells

**DOI:** 10.1111/jcmm.16404

**Published:** 2021-03-08

**Authors:** Simon Trapp, Ali A. Aghdassi, Juliane Glaubitz, Matthias Sendler, Frank Ulrich Weiss, Jens Peter Kühn, Marie‐Luise Kromrey, Ujjwal M. Mahajan, Petra Pallagi, Zoltán Rakonczay, Viktória Venglovecz, Markus M. Lerch, Peter Hegyi, Julia Mayerle

**Affiliations:** ^1^ Department of Medicine A University Medicine Greifswald Greifswald Germany; ^2^ Institute of Diagnostic Radiology and Neuroradiology University Medicine Greifswald Greifswald Germany; ^3^ Department of Medicine II Ludwig‐Maximilians University Munich Munich Germany; ^4^ First Department of Medicine University of Szeged Szeged Hungary; ^5^ Department of Pathophysiology University of Szeged Szeged Hungary; ^6^ Department of Pharmacology and Pharmacotherapy University of Szeged Szeged Hungary; ^7^ Department of Translational Medicine/First Department of Medicine Medical School Institute for Translational Medicine Pécs Hungary

**Keywords:** acute pancreatitis, CFTR, ductal cells, inflammatory cells

## Abstract

Mutations in the cystic fibrosis transmembrane conductance regulator gene (*CFTR*) are an established risk factor for cystic fibrosis (CF) and chronic pancreatitis. Whereas patients with CF usually develop complete exocrine pancreatic insufficiency, pancreatitis patients with *CFTR* mutations have mostly preserved exocrine pancreatic function. We therefore used a strain of transgenic mice with significant residual CFTR function (CFTR^tm1HGU^) to induce pancreatitis experimentally by serial caerulein injections. Protease activation and necrosis were investigated in isolated acini, disease severity over 24h, pancreatic function by MRI, isolated duct stimulation and faecal chymotrypsin, and leucocyte function by ex vivo lipopolysaccharide (LPS) stimulation. Pancreatic and lung injury were more severe in CFTR^tm1HGU^ but intrapancreatic trypsin and serum enzyme activities higher than in wild‐type controls only at 8h, a time interval previously attributed to leucocyte infiltration. CCK‐induced trypsin activation and necrosis in acini from CFTR^tm1HGU^ did not differ from controls. Fluid and bicarbonate secretion were greatly impaired, whereas faecal chymotrypsin remained unchanged. LPS stimulation of splenocytes from CFTR^tm1HGU^ resulted in increased INF‐γ and IL‐6, but decreased IL‐10 secretion. CFTR mutations that preserve residual pancreatic function significantly increase the severity of experimental pancreatitis—mostly via impairing duct cell function and a shift towards a pro‐inflammatory phenotype, not by rendering acinar cells more susceptible to pathological stimuli.

## 1 INTRODUCTION

Pancreatitis is a common disease, often leading to hospital admission ^1^ and its underlying genetic susceptibilities are increasingly recognized,^2^ particularly in patients with chronic or relapsing forms of pancreatitis. Although most pathophysiological studies have focused on acinar cells or acinar cell‐specific events such as a pathological secretagogue response,^3^ the balance between digestive protease activation and degradation via secretory^4^ or lysosomal enzymes,^5^, ^6^, ^7^, ^8^ and the role of acinar cells as the initial site of injury,^9^ the role of other cell types such as duct cells^10^, ^11^ and immune cells^12^, ^13^, ^14^ has only recently become apparent. The cystic fibrosis transmembrane conductance regulator (CFTR) is an ABC transporter‐class ion channel protein that conducts chloride ions across epithelial cell membranes. Loss‐of‐function mutations in the *CFTR* gene were originally discovered to be associated with cystic fibrosis (CF) in affected patients^15^ and later found to play a role in male vas deference infertility and chronic pancreatitis. The latter association was initially detected using the original CF‐mutation panels,^16^, ^17^ subsequently characterized by full exome sequencing, and found to increase the susceptibility for pancreatitis around 2.5‐fold to threefold.^18^, ^19^ It later became obvious that, unlike in CF, mainly patients with exocrine pancreatic sufficiency and carriers of CFTR mutations with maintained residual CFTR function develop pancreatitis.^20^ The question whether and why only some carriers of the more than 1700 known *CFTR* mutations (around 15% of the population in Western countries carry *CFTR* mutations) develop pancreatitis, and which cell types known to contribute to the disease pathogenesis are predominantly affected by CFTR impairment, is still under debate.^21^ It is further complicated by the fact that not only the type of mutation,^22^ but also a number of pleotropic and modifier effects^23^ appear to determine the manner and severity by which the pancreas is involved. Elegant experimental studies that were undertaken to elucidate the underlying mechanism of *CFTR* mutation‐associated pancreatic damage have either used mice with a deleted *cftr*‐gene (null mice) and thus no residual function ^24^ or with transgenically expressed *cftr*‐deletion mutations also causing absence of cftr activity.^25^, ^26^ To experimentally recreate a situation closer to the human geno‐ and phenotype, we have studied experimental pancreatitis in a mouse strain (CFTR^tm1HGU^) with significant residual CFTR function and no spontaneous pancreatic phenotype before adulthood.^27^ Pancreatic enzyme output in these mice was found to be unimpaired, but ductal bicarbonate and fluid secretion were strongly reduced. The severity of the disease was significantly increased, whereas the response and injury of isolated acinar cell remained unaffected. In vitro inflammatory cells from CFTR^tm1HGU^ mice were found to respond to lipopolysaccharide (LPS) with an increased release of pro‐inflammatory, and a decreased release of anti‐inflammatory cytokines. These data indicate that “mild” CFTR mutations with significant residual exocrine pancreatic function increase the susceptibility towards pancreatitis, as well as the disease severity. This occurs independently of acinar cell events, but is due to an impairment of CFTR function in pancreatic duct cells and the immune system.

## 2 MATERIALS AND METHODS

### 2.1 Materials

Caerulein, the biologically active, phosphorylated cholecystokinin (CCK) octapeptide (Tyr(SO 3 H)27)‐cholecystokinin fragment) and amiloride were obtained from Sigma‐Aldrich (Eppelheim, Germany), collagenase (Clostridium histolyticum, EC.3.4.24.3) from SERVA (lot no. 14007), human myeloperoxidase (MPO) from Calbiochem, the substrates R110‐(Ile‐Pro‐Arg) and R110‐(Ala)_4_ from Invitrogen, CBA mouse inflammation kit from Becton Dickinson, ‘Amyl’ amylase quantification kit and ‘Lip’ lipase quantification kit from Roche (Grenzach‐Whylen), forskolin from Tocris Cookson and H_2_DIDS from Invitrogen Corporation. All other chemicals were of highest purity and obtained either from Sigma‐Aldrich, Merck, Amersham Pharmacia Biotech or Bio‐Rad.

### 2.2 Induction of acute pancreatitis in wild‐type and CFTR transgenic mice

**FIGURE 1:**
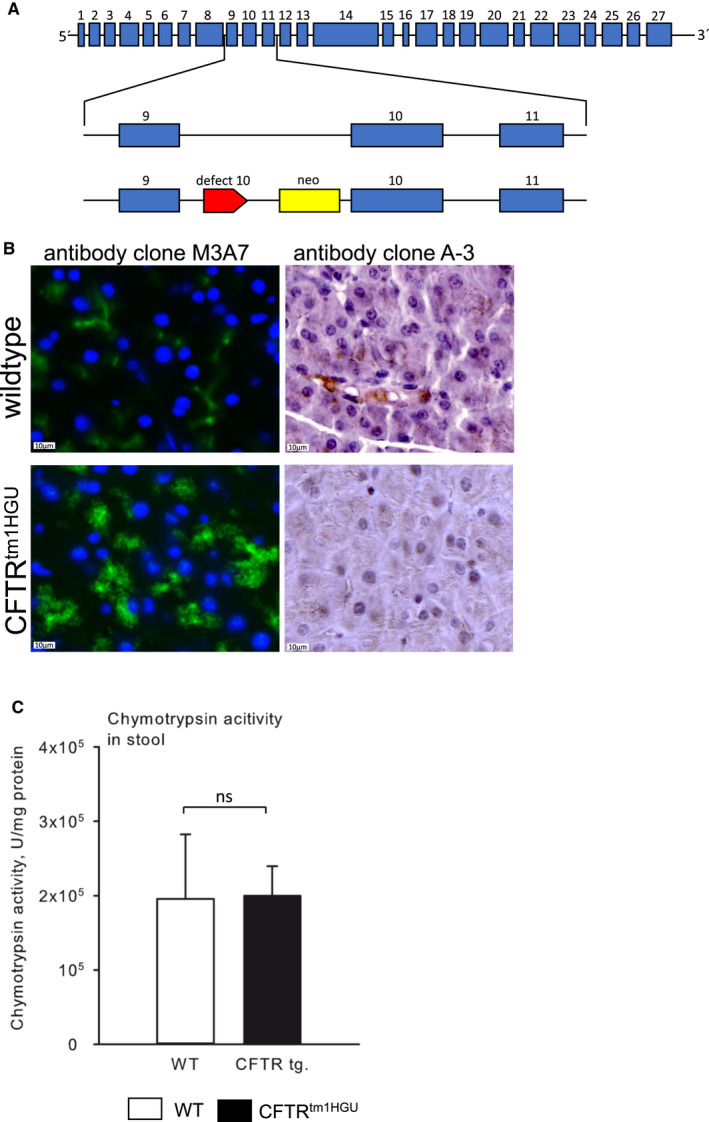
CFTR is aberrantly expressed in acinar cells of CFTR^tm1HGU^ mice, but exocrine pancreatic function is maintained. (A) A transgenic mouse model containing a neomycin cassette and a part of exon 10 of the *CFTR* gene was used that has residual CFTR expression. (B) Immunofluorescence labelling of CFTR (Clone: M3A7, DLN‐06996, Dianova) in wild‐type and CFTR^tm1HGU^ mice showed a clear localization of CFTR on the cellular membrane in wild‐types and a prominent labelling in the cytoplasm of CFTR^tm1HGU^ mice. A second antibody (Clone: A‐3, Santa Cruz Biotechnology) was used for immunohistochemistry to verify the labelling. We confirmed the localization of CFTR at the cell membrane in wild‐type mice, which was absent in CFTR^tm1HGU^ animals. (C) Comparable stool chymotrypsin activities wild‐type and CFTR^tm1HGU^ mice indicate that exocrine pancreatic enzyme secretion is not impaired in the transgenic animals

All animal experiments were carried out after prior approval by the institutional animal care committee. CFTR^tm1HGU^ mice were originally generated by Dorin et al^28^ and kindly provided by the colleagues B. Tümmler and U. Seidler from the Medical University of Hannover (MHH). By using an insertional vector that encompasses a part of intron 9 and extends into exon 10 (Figure 1A), the *CFTR* gene is disrupted and offspring of heterozygous mice were crossed leading to a phenotype with an impaired chloride ion transport but significant residual CFTR function of over 20% because the gene disruption by insertion does not result in the loss of any genomic sequence and the mutation is ‘leaky’ allowing for aberrant splicing of significant wild‐type *cftr* mRNA.^27^ These mice have no spontaneous pancreatic phenotype and good long‐term survival.^29^ Wild‐type littermates were used as controls. All animals were maintained according to institutional guidelines and protocols of the local animal facility and approved by the institutional animal care and use committee. For this study, 14‐week‐old male animals were used. Mice were starved overnight with access to water ad libitum. Acute pancreatitis was induced by 8 hourly intraperitoneal injections of CCK/caerulein (50 µg/kg bodyweight) as previously reported.^30^

### 2.3 Preparation of serum and tissue samples

After kill, serum was aliquoted, snap‐frozen and stored at −20°C for further analysis. Pancreatic tissue was partitioned and either frozen in liquid nitrogen and stored at −80°C for later enzymatic measurements, fixed in 5% paraformaldehyde for paraffin embedding, or put into Cryomold ® embedding folds (Tissue Tek, Sakura Finetek) and snap‐frozen. Parts of tissue used later for enzymatic assays were homogenized on ice in a buffer consisting of 100 mmol/L Tris, 5 mmol/L CaCl_2_ and pH 8.0.

### 2.4 Preparation of pancreatic acinar cells

Acinar cells were prepared by collagenase digestion and isolated as previously described.^4^, ^31^ Cells were maintained in Dulbecco's modified Eagle medium containing 2% bovine serum albumin (BSA) and 10 mmol/L 4‐(2‐hydroxyethyl)‐1‐piperazine ethanesulphonic acid (HEPES) and stimulated with 1 μmol/L CCK. Intracellular elastase activity was measured for up to 1 hour using the substrate R110‐CBZ‐(Ala)_4_ in a fluorescence plate reader (BMG Labtech, Ortenberg, Germany). Cellular necrosis was measured by propidium iodine exclusion.^32^ All measurements were done in triplicates in a 96‐well microtitre plate.

### 2.5 Isolation of pancreatic ducts

Pancreatic ducts were isolated using a protocol as described previously.^33^ Intra‐ and interlobular ducts were isolated by enzymatic digestion and microdissection. Isolated ducts were incubated overnight. During the overnight incubation, the ends of the ducts seal and begin to swell due to fluid secretion of the ductal cells.

### 2.6 Isolation of splenocytes

Leucocytes were isolated from spleens of CFTR^tm1HGU^ and wild‐type mice using a 70‐µm nylon cell filter (BD Falcon) to remove aggregates and cell debris.^34^ Erythrocytes were lysed by a solution containing 155 mmol/L NH_4_Cl, 10 mmol/L KHCO_3_ and 0.1 mmol/L EDTA. Neutrophil granulocytes were isolated from total leucocytes using a MACS® cell separation kit according to the manufacturer's instruction (Miltenyi Biotech, Bergisch‐Gladbach, Germany).^35^, ^36^ For separation of neutrophils, Ly6g served as a specific antibody. Both splenocytes and neutrophil granulocytes were counted in a Neubauer chamber and transferred to sterile PBS.

### 2.7 Biochemical assays

Activity of serum lipase and amylase was measured by photometric absorbance assays (Roche Hitachi, Grenzach‐Whylen, Germany) as kinetics over 30 min with an absorbance at 570 nm at 37°C. Purified enzymes (Sigma) were used for standardization. Trypsin activity in pancreatic homogenates was measured fluorometrically at 37°C for 1 hour using the substrate R110‐CBZ‐Ile‐Pro‐Arg (Invitrogen, Karlsruhe, Germany). Protein amount was quantified using the Bradford assay. To determine exocrine enzyme secretion, we measured chymotrypsin activity in stool as previously reported.^37^ Briefly, stool was collected, stored at −20°C and later samples of 15‐30 mg suspended in solvent (0.1% Triton X‐100, 0.5 M NaCl and 100 mmol/L CaCl_2_), briefly sonicated and centrifuged at 20,000 r.p.m. for 10 minutes at 0°C. Chymotrypsin activity was determined flurometrically using 5 µmol/L AMC‐(Suc‐Ala_2_‐Pro‐Phe) (Bachem) and calculated as U mg^‐1^ with purified chymotrypsin as internal standard and activities expressed in relation to faecal weight. MPO activity was measured as previously reported.^38^ Briefly, pancreatic tissue was homogenized on ice in a 20 mmol/L potassium phosphate buffer (pH 7.4) and centrifuged. The pellet was suspended in a buffer containing 50 mmol/L potassium phosphate (pH 6.0) and 0.5% cetyltrimethylammonium bromide. MPO activity was measured using 0.53 mmol/L O‐dianisidine and 0.15 mmol/L H_2_O_2_ as substrates and SpectraMax Spectrophotometer (Molecular Devices). The results are calculated as fold change in comparison to unstimulated wild‐type animals.

For measurement of cytokine secretion, splenocytes were stimulated with 1 μg/mL LPS as reported ^12^, ^32^, ^36^ and activity in the supernatants was quantified with the CBA mouse inflammation kit following the manufacturer's instructions (Becton Dickinson) and fluorescence activated cell sorting (FACS).

### 2.8 Histology, immunohistochemistry and immunofluorescence staining of pancreatic tissues

Paraffin embedded tissues were sliced and stained with haematoxylin and eosin. For each animal, ten randomly chosen microscopic fields were investigated under a light microscope. For quantification of oedema, necrosis and leucocyte infiltration, a modified histopathologic score adapted from Kyogoku et al ^39^ was used, that scored 0 for absent, 1 for less than 20%, 2 for 20%‐50% and 3 for more than 50% as previously described.^40^ Immunohistochemistry was performed from paraffin embedded tissue samples as previously reported. The following primary antibodies were used in a dilution of 1 to 200: anti‐CFTR (Clone: A‐3, Santa Cruz Biotechnology, sc‐376683), p67‐phox (Santa Cruz Biotechnology, sc‐374510) and anti‐CD11b (abcam, ab133357). For immunofluorescence staining of CFTR snap‐frozen tissue embedded with Tissue‐Tek® OCT^TM^ compound (Sakura Finetek, Alphen aan den Rijn) was cut into 1‐ to 2‐µm‐thick slices, fixed with 20% acetone and washed with PBS. The anti‐CFTR antibody (Clone: M3A7, DLN‐06996, Dianova) was used in a 1:100 dilution in 20% FCS, and incubation was performed over night at 4°C. An anti‐rat IgG FITC‐labelled antibody (Jackson ImmunoResearch) served as a secondary antibody. Nuclei were stained by DAPI and slides were mounted with DACO mounting medium (Agilent Technologies Inc) for immunofluorescence.

### 2.9 Measurement of intracellular pH

Intracellular pH (pH_i_) was estimated using techniques as described before.^41^ Briefly, isolated ducts were bathed in standard HEPES solution at 37°C and were loaded with the membrane‐permeable acetoxymethyl derivative of BCECF (2 μmol/L) for 20‐30 minutes. After loading, the ducts were continuously perfused with solutions at a rate of 5‐6 mL/min. pH_i_ was measured using a CellR imaging system. Four to seven regions of interest (ROIs) of 20‐28 cells in each intact duct were excited with light at wavelengths of 490 and 440 nm, and the 490/440 fluorescence emission ratio was measured at 535 nm.^33^

### 2.10 Measurement of HCO_3_^‐^ secretion

We used the inhibitory stop method to determine the HCO_3_^‐^ efflux across the luminal membrane: Basolateral Na^+^/HCO_3_^‐^ co‐transporters, Cl^‐^/HCO_3_^‐^ and Na^+^/H^+^ exchangers were blocked using H_2_DIDS (0.5 mmol/L) and amiloride (0.2 mmol/L) administered from the basolateral side of ductal cells for 5 minutes. The effects of H_2_DIDS and amiloride are likely to be confined to the basolateral transporters because a) H_2_DIDS is unlikely to gain rapid access to the lumen of the sealed ducts because of its charged sulphonic acid groups, and b) there are no amiloride‐sensitive transporters at the apical membrane of small interlobular ducts. The inhibition of basolateral transporters caused a marked decrease in pH_i_. The rate of pH_i_ acidification after H_2_DIDS and amiloride exposure reflects the intracellular buffering capacity and the rate at which HCO_3_^‐^ efflux is secreted across the luminal membrane via Cl^‐^/HCO_3_^‐^ exchangers and possibly CFTR.^42^ The initial rate of intracellular acidification (dpH/dt) over the first 60 seconds starting from the administration of inhibitors was calculated by linear regression analysis using 60 data points (one pH_i_ measurement per second).

### 2.11 Measurement of in vitro fluid secretion

Fluid secretion into the closed luminal space of the cultured ducts was analysed using a swelling method developed by Fernandez‐Salazar et al^43^ Briefly, the ducts were transferred to a perfusion chamber and perfused with either standard HEPES or the HCO_3_^−^/CO_2_‐buffered solution at 37°C. Bright‐field images were acquired at 1‐min intervals using a CCD camera (CFW 1308C, Scion Corporation). Digital images of the ducts were analysed using Scion Image software (Scion Corp.) to obtain values for the area corresponding to the luminal space in each image. Luminal area measurements from individual images were normalized to the average of the first few in the series (A0) thus giving values for the relative area (AR = A/A0). These were then converted to relative volumes (VR = V/V0) by assuming that the lumen was cylindrical and by taking into account the relative increases in width and length in each series. Secretory rates were calculated from the rate of change of luminal volume and were normalized to the luminal epithelial surface area. Secretory rate was expressed in picolitres per minute per square millimetre for the last 20 minutes of forskolin stimulation.

### 2.12 Measurement of in vivo fluid secretion

Small animal magnetic resonance imaging (MRI) was used to determine stimulated pancreatic fluid output as previously reported ^44^ and wild‐type controls compared to CFTR^tm1HGU^ animals. Animals were allowed free access to pineapple juice 12 hours before the MRI examination. MRI was performed in a 7.1 Tesla animal scanner (Bruker). Strong T2‐weighted series of the complete abdomen were acquired before and after retroorbital injection of 10 IU units/kg body weight (b.w.) secretin (ChiroStim, ChiRhoClin, Burtonville, MD, United States). The time between injection and MRI was 6 min. The sequences were acquired using the image parameters: TR/TE 4400/83 ms; flip angle: 180°; matrix 256 × 256; field of view 40 × 40 mm; bandwidth 315 Hz/pixel; slice thickness 1 mm; 20 slices. All image analyses were performed using Osirix (version 5; Pixameo, Bernex). In order to exclude effects of the basal secretion, MRI datasets before were subtracted from datasets after stimulation. The created images show the total excretion after secretin stimulation. Excreted fluid is defined as high signal intensity in created images. The fluid excretion into the small intestine was segmented in each slice. The software calculated the volume of the segmented areas as total excreted volume (TEV).

### 2.13 Statistical analysis

The collected data are shown as means ± SEM from at least five animals per time point and group. Statistical analysis was performed by SigmaPlot (Systat Software GmbH, Erkrath, Germany) using the Students t‐test for independent samples. The data from the pancreatic duct experiments were analysed by one‐ or two‐way ANOVA tests.

## 3 RESULTS

### 3.1 Characterization of the CFTR^tm1HGU^ mouse model

The insertion mutation (CFTR^tm1HGU^) between exons 9 and 10 (Figure 1A) disrupts CFTR function but allows for significant amounts (median 37% of mRNA transcript) of alternatively spliced wild‐type *cftr* mRNA to be transcribed. When we localized CFTR in the exocrine pancreas, we detected it as previously reported^45^ in duct cells as well as at the luminal membrane of acinar cells in wild‐type animals (Figure 1B upper panel). In the untreated pancreas of CFTR^Rtm1HGU^ animals, the subcellular localization was identical, but a significant increase of fluorescent label was found in the cytosol of acinar cells, as opposed to being strictly confined to the cell membrane (Figure 1B lower panel). The usage of a second anti‐CFTR antibody (from Santa Cruz) confirmed these finding by immunochemistry and indicated a decreased membrane localization of the CFTR protein (Figure 1B antibody clone A‐3). To determine whether or not CFTR modification had affected the output of exocrine pancreatic enzymes, we compared the activity of chymotrypsin in stool pellets from CFTR^Rtm1HGU^ animals and wild‐type littermates and found no significant difference (Figure 1C). This indicates that CFTR^Rtm1HGU^ mice have no exocrine pancreatic enzyme insufficiency.

### 3.2 Increased severity of acute pancreatitis in CFTR^tm1HGU^ mice

**FIGURE 2:**
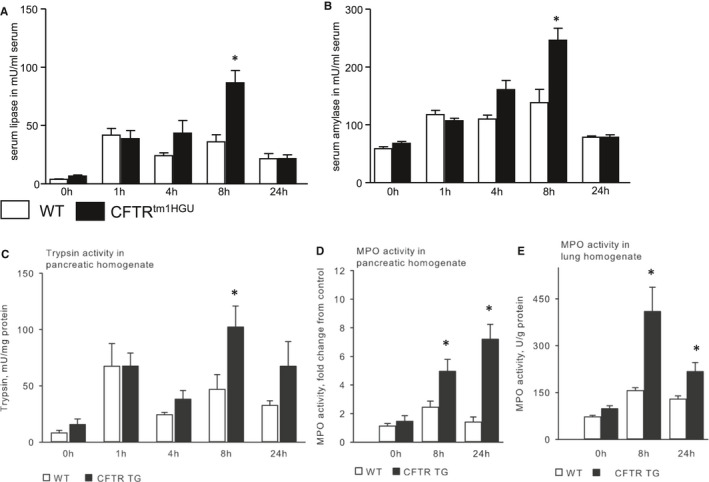
Acute pancreatitis has a more severe course in CFTR^tm1HGU^ mice. (A and B) Acute pancreatitis was induced in wild‐type and CFTR^tm1HGU^ mouse strains by intraperitoneal injections of caerulein (8 x50 µg/kg bodyweight). Serum amylase and lipase activities were higher in CFTR^tm1HGU^ mice only at 8h, but not after 1h, a time point directly affected by the pathological stimulation of acinar cells. (C) During acute pancreatitis, trypsin shows a typical biphasic curve of its activity after 1 and 8 h. There was no significant difference in activity between both groups at 1 h. However, at 8 h trypsin activity was increased in the transgenic mice. (D, E) Local infiltration of neutrophils into the pancreas and lungs was determined by measuring MPO activity in tissue homogenates. At 8 and 24 h after onset of the pancreatitis the activity was markedly increased in CFTR^tm1HGU^ mice

To investigate whether a modified CFTR expression alters disease severity, we induced acute pancreatitis in CFTR^tm1HGU^ mice and corresponding wild‐type animals by repeated intraperitoneal caerulein injections. During the first 24 hours, the CFTR transgenic mice clearly developed a more severe disease course. In all animals, serum amylase, lipase as well as intrapancreatic trypsin activities increased after induction of pancreatitis. As previously shown,^46^ these enzyme activities showed a biphasic curve with peaks at one and 8 hours of which the first peak is thought to be induced by direct secretagogue effects on acinar cells, whereas the second is thought to be mediated by activated inflammatory cells. Interestingly, only the later, second peaks were greater in CFTR^tm1HGU^ animals when compared to their wild‐type controls with pancreatitis, suggesting the direct response to supramaximal CCK stimulation of acinar cells did not differ from controls in the pancreas of CFTR‐disrupted animals (Figure 2A‐C). MPO activity, a surrogate marker for leucocyte infiltration, was higher in CFTR^tm1HGU^ animals than in controls, and it peaked at 24h in the pancreas and at 8h in the lungs (Figure 2D&E).

**FIGURE 3:**
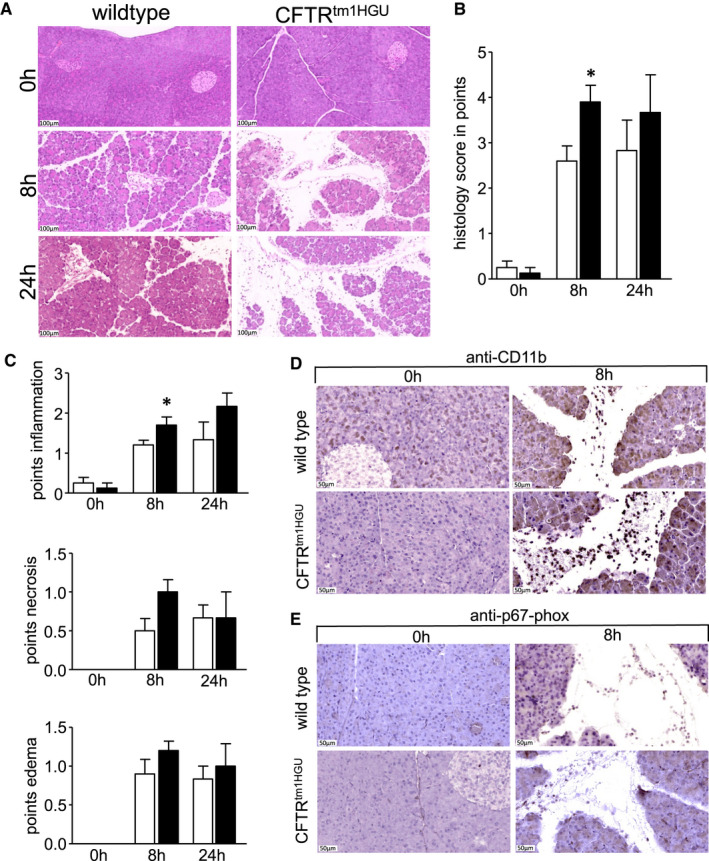
Histological damage was increased in CFTR transgenic mice as shown by haematoxylin and eosin stainings (A). The calibration bar represents 100 µm. (A) Quantification of histological changes was performed by a separate evaluation of necrosis, inflammation and pancreatic oedema (B). Summed up, there was a significant increase of pancreatic damage at 8h after induction of pancreatitis in the CFTR transgenic animals, seen by a higher histology score (B). The single parameters showed a clear trend towards an increase but only the quantification of the infiltrating leucocytes reached significance 8h after the onset of the disease (C), and still showed a trend at 24 h. A detailed analysis of infiltrating leucocytes by immunohistochemical staining of CD11b and p67‐phox showed a prominent infiltration of CD11b + macrophages in both mice strains at 8h after induction of pancreatitis that was more predominant in the CFTR^tm1HGU^ mice (D). In contrast to CD11b + macrophages, p67‐phox‐positive neutrophils could rarely be observed (E). At least five mice were used in every group. The experiments were performed in triplicates. Asterisks indicate significant differences with *P* <.05

On histology, the pancreas of CFTR^tm1HGU^ mice clearly showed no signs of damage in the absence of caerulein stimulation, whereas all animals developed cellular vacuolization, interstitial oedema, tissue necrosis and inflammatory infiltrates in response to disease induction (Figure 3A). Tissue injury was clearly more prominent in CFTR^tm1HGU^ animals and when we scored and quantitated the results for histology score, which was most pronounced 8h after disease onset of pancreatitis (Figure 3B). A detailed examination of individual parameters of the histology score showed a significant increase of inflammatory infiltrate and a trend towards increased oedema and necrosis at 8h in the CFTR^tm1HGU^ mice (Figure 3C). Immunohistochemical stainings of macrophages by anti‐CD11b (Figure 3D) and neutrophils by anti‐p67‐phox (Figure 3E) in pancreatic tissue samples indicated in both wild‐type and CFTR^tm1HGU^ animals that CD11b + macrophages represent the dominant infiltrating immune cell type, whereas p67‐phox positive neutrophils ^34^ could only be detected sporadically. In CFTR^tm1HGU^ animals there was a markedly stronger infiltration of CD11b + macrophages, which could explain the increased pancreatic damage.

### 3.3 Premature intracellular zymogen activation and cell injury in CFTR^tm1HGU^ mice

To test whether the absence of a greater disease severity after one hour of pancreatitis was due to an acinar cell‐specific effect in CFTR^tm1HGU^ mice, we isolated acini from the pancreas of CFTR‐disrupted and wild‐type animals and incubated them in parallel with CCK for up to 60 minutes.

**FIGURE 4:**
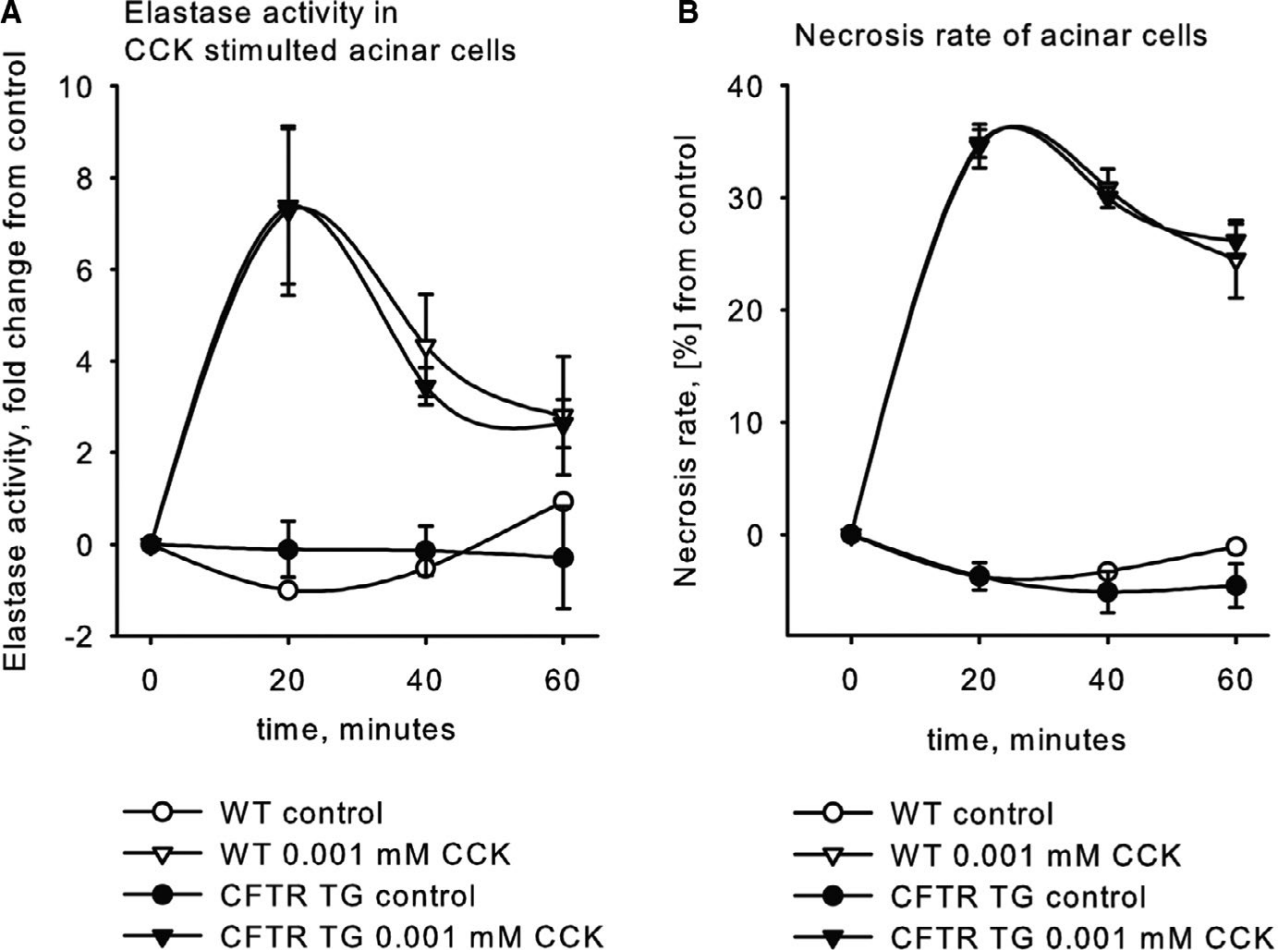
Premature intracellular zymogen activation and necrosis are similar in CFTR‐mutant and wild‐type mice. (A) Intracellular elastase activation in CFTR^tm1HGU^ mice upon supramaximal CCK stimulation was equal to controls. (B) Cellular necrosis measured by inclusion of propidium iodide was not affected by the gene mutation. Data points show means ± SE of at least 5 experiments in each group and each time point

Although both, wild‐type and acini from CFTR^tm1HGU^ mice, showed a rapid increase in intracellular protease activity (Figure 4A) and in acinar cell necrosis (Figure 4B) as indicated by increase propidium‐iodine uptake, there was no significant difference between wild‐type and CFTR^tm1HGU^ animals upon CCK stimulation. This indicates that disruption of CFTR had no measurable effect on premature digestive enzyme activation or acinar cell injury and suggests that other mechanisms and cell types must account for the greater in vivo disease severity in CFTR^tm1HGU^ mice.

### 3.4 Duct cell function in CFTR^tm1HGU^ mice

**FIGURE 5:**
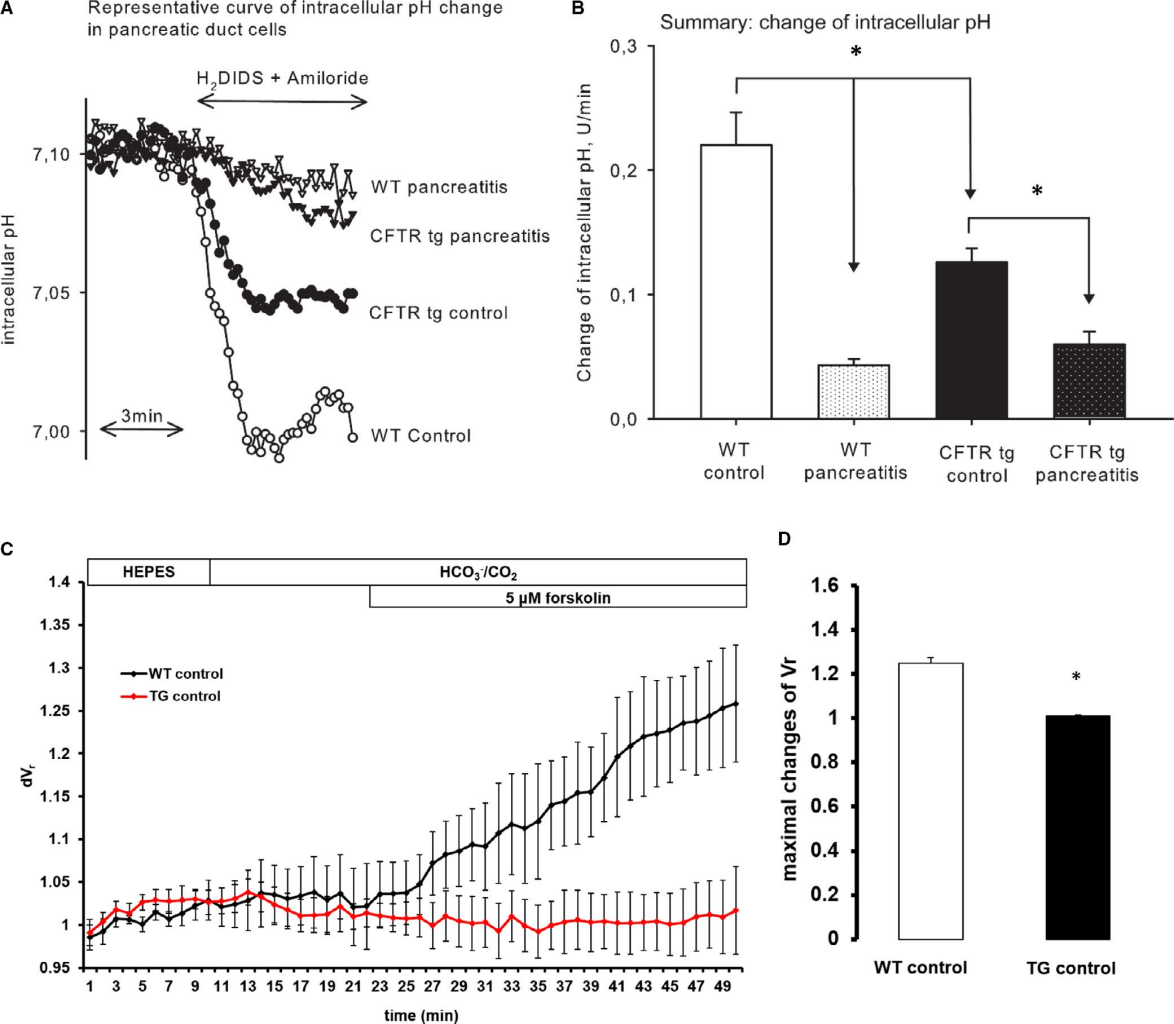
Changes in HCO_3_^‐^ and fluid secretion in pancreatic duct cells in CFTR^tm1HGU^ and wild‐type mice during acute pancreatitis. Intralobular pancreatic ducts were isolated 1 h after the last caerulein injection. The ducts were used for experiments after overnight incubation. (A) Representative intracellular pH (pH_i_) traces of pancreatic duct cells, demonstrating the effect of 0.2 mmol/L amiloride and 0.5 mmol/L H_2_DIDS administered from the basolateral membrane in standard HCO_3_^‐^/CO_2_‐buffered solution. (B) Summarized data for the dpH/dt changes that were calculated by linear regression analysis of pH_i_ measurements made over the first 60 s after exposure of the transport inhibitors. (C) Wild‐type animals clearly responded to forskolin stimulation by increased fluid secretion, but the CFTR^tm1HGU^ animals did not. (D) The maximum of fluid secretion was significantly lower in CFTR^tm1HGU^ compared to wild‐type mice. Pancreatic ducts were prepared for at least from five mice per group and measurements were performed in triplicates. Asterisks indicate significant differences with *P* <.05

Previous studies have shown that a lack of CFTR in pancreatic duct cells leads to decreased bicarbonate secretion and impairs their physiological function.^47^, ^48^ We therefore investigated the degree of duct cell impairment in CFTR^tm1HGU^ mice by quantitating HCO_3_^‐^ secretion using the inhibitory stop method. Exposing isolated ducts to 0.5 mmol/L H_2_DIDS and 0.2 mmol/L amiloride caused intracellular acidosis due to inhibition of the basolateral Na^+^/HCO_3_^‐^ co‐transporters and Na^+^/H^+^ exchangers ^49^ (Figure 5A). The summary of dpH/dt changes after addition of H_2_DIDS and amiloride in the different groups is displayed in Figure 5B. HCO_3_^‐^ secretion was significantly lower in mutant mice compared to wild‐type controls. Secondly, changes of pH_i_ were more overt in ducts from acute pancreatitis animals than from controls but ultimately similar between groups. In the next step, fluid secretion of interlobular pancreatic ducts was determined by using video microscopy to measure the rate of swelling of isolated duct segments that had sealed following overnight culture. Initially, ducts were perfused with standard HEPES‐buffered solution and then perfusion was switched to standard HCO_3_^−^/CO_2_‐buffered solution. Ducts were stimulated with 5 μmol/L forskolin from 10 minutes thereafter. Stimulation of the WT ducts with 5 μmol/L forskolin caused a dynamic swelling of the ducts (Figure 4C) as a result of fluid secretion into the closed luminal space. The secretory rate, calculated from the rate of change in luminal volume, was 211 ± 58 pl/min/mm^2^ after stimulation. In contrast, ducts from TG mice did not show increased secretion in response to forskolin (Figure 5C). The secretory rate was 31 ± 74 pL/min/mm^2^, which represents a reduction of 85% compared with the WT controls. The summary of fluid secretion data is shown in Figure 5D. The maximal changes of relative luminal volume are significantly reduced in mutant mice compared to wild‐type controls.

**FIGURE 6:**
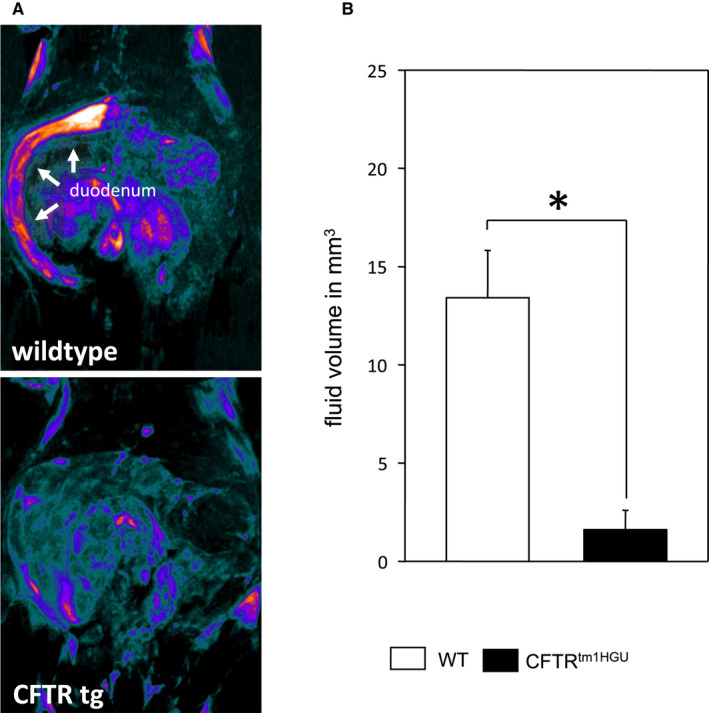
Fluid secretion after secretin stimulation in magnetic resonance cholangiopancreatography (MRCP). Fluid volume was markedly reduced in CFTR^tm1HGU^ compared to wild‐type mice after secretin stimulation (A + B)

To test whether this impairment translates to the in vivo situation, we used Magnetic Resonance Imaging (Figure 6A) of mice stimulated with secretin and found a prominent decrease in fluid secretion in the CFTR^tm1HGU^ mice, which was reduced to one fifth of that in wild‐type controls (Figure 6B). These data demonstrate a strong impairment of pancreatic duct cell function in CFTR^tm1HGU^ mice, which differed greatly from the effects on acinar cells.

### 3.5 Impairment of leucocyte function in CFTR^tm1HGU^ mice

**FIGURE 7:**
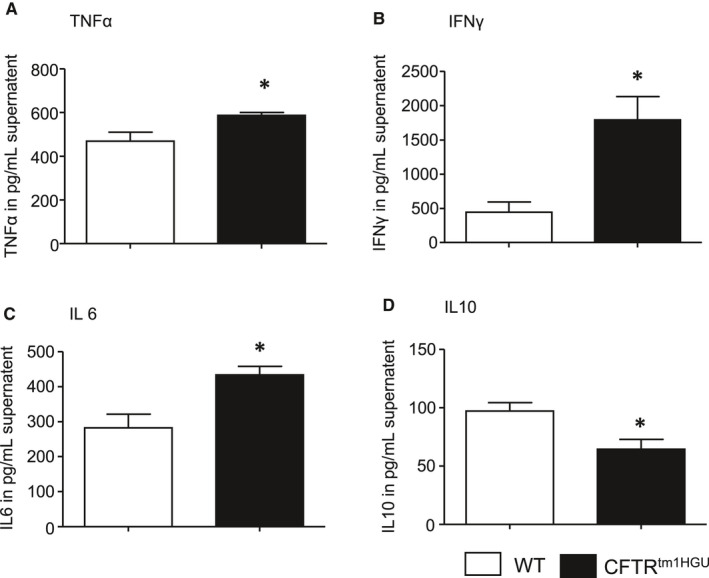
Pro‐inflammatory alterations of the immune system in isolated and LPS stimulated splenocytes of CTFR‐mutant mice. (A‐C) An elevated secretion of TNFα, INF‐γ and IL6 was found, whereas (D) release of the anti‐inflammatory IL‐10 was lower in the supernatant of splenocytes derived from transgenic animals. Splenocytes were isolated from at least five mice per group and measurements were performed in triplicates. Data points show means ± SE Asterisks indicate significant differences with *P* <.05

The differences between CFTR^tm1HGU^ and wild‐type mice in terms of serum pancreatic enzyme and intrapancreatic trypsin activities in the later (rather than the early) disease course of pancreatitis, as well as the differences in MPO activity in the lungs and pancreas of the animals, suggested that the effects of the CFTR transgene are ultimately connected to infiltrating inflammatory cells. These findings prompted us to analyse the response of immune cells from CFTR^tm1HGU^ animals directly. When we stimulated isolated splenocytes with lipopolysaccharide (LPS) and measured the cytokine release, we detected a significantly increased secretion of the pro‐inflammatory cytokines TNF‐α, IFN‐γ and IL‐6 in splenocytes of the transgenic mice (Figure 7A‐C). On the other hand, the anti‐inflammatory cytokine IL‐10 was found to be reduced in the transgenic animals (Figure 7D). This combination of an increased pro‐inflammatory cytokine secretion along with a decreased anti‐inflammatory chemokine release could represent an additional explanation for greater disease severity in CFTR^tm1HGU^ mice.

## 4 DISCUSSION

Mutations in the *CFTR* gene lead to a defective CFTR channel protein, a major regulator of the transcellular transport of chloride and bicarbonate. *CFTR* mutations were originally identified in patients with cystic fibrosis (CF), but later they were also found to be associated with male vas deference infertility and chronic or recurrent pancreatitis. CF is perhaps the best investigated genetic disorder and most frequently based on a loss of the amino acid phenylalanine in codon 508 (delF508), either on both alleles or in combination with other variants of the *cftr*‐gene.^15^ CF patients often show a very variable clinical course including pulmonary complications such as bronchiectasis, increased mucus production and chronic endobronchial inflammation but also an impairment of other organs that include the reproductive system leading to infertility, the liver with abnormal liver function and cirrhosis, and the pancreas with exocrine and endocrine insufficiency.^50^ The variable extent of pancreatitis that develops in patients with CFTR mutations^16^, ^17^, ^18^ is quite distinct from the exocrine pancreatic insufficiency that develops in CF patients and typically arises in subjects without overt impairment of pulmonary function and with preserved exocrine pancreatic function. Sometimes the difference is not quite clear‐cut and subjects who initially presented with pancreatitis alone can still develop pulmonary dysfunction later in life and may have to be reclassified as mild cases of CF.^51^ Whether the pancreatitis‐only phenotype is due to mutations (or compound heterozygous combinations of mutations) that confer a milder defect in CFTR function or whether pleotropic and modifier effects determine the pancreatitis phenotype is still being debated.^23^ Some attempts have been made to elucidate the underlying mechanism of pancreatitis under impairment of the CFTR experimentally. They either used mice with complete deletion of the *cftr*‐gene ^24^ or transgenic animals carrying the most common deletion mutant found in humans with CF.^25^ The acinar cell defects identified in these animals included trafficking defects of zymogen granule membranes from the lumen,^52^ not unlike those found in models of biliary pancreatitis,^53^ solubilization of secretory (pro)enzymes, dilatation of the acinar lumen, a reduction in zymogen granules and persistent aggregation of secretory enzymes released into the luminal space. Another study found greater acinar cell injury in response to supraphysiological stimulation, a more severe inflammatory response and, most prominently, reduced apoptosis.^26^ Some of these effects are clearly consistent with pancreatitis und are compatible with pancreatic manifestation of CF.

To experimentally create a situation that mimics the human situation of pancreatitis in the presence of preserved residual CFTR function and pancreatic sufficiency more closely, we used a previously reported strain of mice (CFTR^tm1HGU^).^28^ These mice have a normal life expectancy and no obvious pancreas phenotype,^27^ which we confirmed by a regular pancreatic microanatomy in untreated transgenic animals. Moreover, exocrine pancreatic function, as determined by faecal excretion of chymotrypsin, was also found to be unimpaired, whereas it is known to be defective in other genetic disorders of the pancreas.^37^ At early time intervals of pancreatitis, which are considered hallmarks of acinar cell damage induced by the secretagogue, we found that serum pancreatic enzymes and intrapancreatic trypsinogen activation did not differ between CFTR^tm1HGU^ and wild‐type controls. When we tried to reproduce this effect (or better the absence of this acinar cell effect) in isolated acini ex vivo, we again found no difference in the response to injury between the CFTR^tm1HGU^ and control mice.

Yet, pancreatitis in vivo was clearly more severe in terms of histological overall damage to the pancreas and specifically in terms of leucocyte infiltration of the pancreas and lungs.

One part of the organ that has clearly moved into the focus of pancreatitis researchers^11^ as well as the CFTR community^54^ is the pancreatic duct. Here, our data were much more consistent with the impairment of bicarbonate and fluid secretion as well as pH_i_ regulation as it would be expected from a modification in *CFTR* gene affecting function. All were found to be significantly impaired in isolated pancreatic ducts as well as in vivo on MRI‐imaging and fluid quantitation. In the absence of an immediate or direct acinar cell effect, this finding readily explains the greater severity of pancreatitis.

A third reason of greater severity would be a change in inflammatory cell function. Duct occluding neutrophils and their extracellular traps,^14^ inflammatory cells cleaving the junctions between acinar cells ^7^, ^13^, ^34^ or directly inducing digestive protease activation by releasing TNFα^36^ are well‐established mechanisms of pancreatitis induction. The majority of infiltrating cells are CD11b + macrophages, which is a clear sign of a greater severity, because infiltrating macrophages directly correlate with the disease severity and local pancreatic damage ^32^, ^36^ as well as systemic immune response.^12^ Macrophages phagocytose necrotic cells and polarize into a pro‐inflammatory M1‐like phenotype, leading to a massive release of pro‐inflammatory cytokines such as IL‐1β, IL‐6, IL‐18 and TNFα, which contribute to local and systemic injury.^12^ Mutations in *CFTR* have also been reported to mediate effects on cells of the immune system.^55^, ^56^ When we found the infiltration of immune cells into the lungs and pancreas during pancreatitis to be a most prominent feature of greater severity in the CFTR^tm1HGU^ animals, we investigated whether the same effect could be reproduced ex vivo in isolated splenocytes. Here, we found that LPS stimulation resulted in an increased release of pro‐inflammatory INFγ, IL‐6 and TNFα and a reduced release of the anti‐inflammatory IL‐10 into the supernatant. These observations are in line with results from other groups who found an increased release of pro‐inflammatory cytokines in CFTR‐deleted or downregulated epithelial cells, mediated by increased p65/NFκB activation,^57^ which also plays an important role in pancreatic acinar cells ^58^ and infiltrating macrophages.^32^ This observation clearly indicates that *CFTR* mutations, even those that leave acinar cells in experimental pancreatitis unaffected, may have a significant effect on the immune system. This may also have contributed to the greater severity of pancreatitis. Whether the altered immune response of CFTR^tm1HGU^ mice is a direct effect of CFTR impairment in the cell membrane of immune cells or, rather, an indirect effect mediated by tissue‐resident immune cells in organs such as the intestine, which are, unlike the exocrine pancreas, directly affected by the CFTR^tm1HGU^ transgene,^27^ cannot be answered conclusively at this point.

In conclusion, our results confirm that impairment of CFTR function increases the susceptibility of the pancreas towards exogenous injury and clearly renders the disease course of acute pancreatitis more severe. In an experimental model in which sufficient residual CFTR function is maintained and animals have not developed exocrine pancreatic insufficiency, the underlying mechanisms appear to involve duct cells and the immune system, whereas acinar cells are not the source but a target of the CFTR pancreas phenotype.

## CONFLICT OF INTEREST

The authors confirm that there are no conflicts of interest.

## AUTHOR CONTRIBUTIONS

**Simon Trapp:** Data curation (equal); Formal analysis (equal); Investigation (equal); Validation (equal); Visualization (equal); Writing‐original draft (equal); Writing‐review & editing (equal). **Ali Aghdassi:** Data curation (equal); Formal analysis (equal); Project administration (equal); Validation (equal); Visualization (equal); Writing‐original draft (equal); Writing‐review & editing (equal). **Juliane Glaubitz:** Data curation (equal); Investigation (equal); Validation (equal); Writing‐review & editing (equal). **Matthias Sendler:** Conceptualization (equal); Data curation (equal); Investigation (equal); Project administration (equal); Validation (equal); Visualization (equal); Writing‐original draft (equal); Writing‐review & editing (equal). **Frank Ulrich Weiss:** Conceptualization; Formal analysis; Methodology (equal); Project administration; Supervision (equal); Writing‐review & editing. **Jens Peter Kühn:** Data curation (equal); Formal analysis (equal); Investigation (equal); Validation (equal); Visualization (equal); Writing‐review & editing (equal). **Marie‐Luise Kromrey:** Validation (equal); Visualization (equal); Writing‐review & editing (equal). **Ujjwal Mukund Mahajan:** Formal analysis (equal); Investigation (equal); Supervision (equal); Validation (equal); Visualization (equal); Writing‐review & editing (equal). **Petra Pallagi:** Data curation (equal); Formal analysis (equal); Methodology (equal); Supervision (equal); Validation (equal); Writing‐review & editing (equal). **Zoltán Rakonczay:** Data curation (equal); Formal analysis (equal); Investigation (equal); Supervision (equal); Validation (equal); Writing‐review & editing (equal). **Viktória Venglovecz:** Data curation (equal); Formal analysis (equal); Investigation (equal); Methodology (equal); Supervision (equal); Validation (equal); Writing‐review & editing (equal). **Markus Lerch:** Conceptualization (equal); Funding acquisition (equal); Methodology (equal); Project administration (equal); Supervision (equal); Writing‐original draft (equal); Writing‐review & editing (equal). **Peter Hegyi:** Conceptualization (equal); Formal analysis (equal); Methodology (equal); Supervision (equal); Validation (equal); Writing‐original draft (equal); Writing‐review & editing (equal). **Julia Mayerle:** Conceptualization (equal); Data curation (equal); Funding acquisition (equal); Methodology (equal); Project administration (equal); Supervision (equal); Validation (equal); Writing‐original draft (equal); Writing‐review & editing (equal).

## Data Availability

The data that support the findings of this study are available from the corresponding author upon reasonable request.
